# Management of hydrocephalus in patients with leptomeningeal metastases: an ethical approach to decision-making

**DOI:** 10.1007/s11060-018-2949-7

**Published:** 2018-07-18

**Authors:** Nayan Lamba, Tim Fick, Rhishi Nandoe Tewarie, Marike L. Broekman

**Affiliations:** 1 Department of Neurosurgery, Computational Neurosciences Outcomes Center, Brigham and Women’s Hospital, Boston, MA USA; 2 Harvard Medical School, Boston, MA USA; 3 Department of Neurosurgery, Brain Center Rudolf Magnus, University Medical Center Utrecht, Utrecht, The Netherlands; 4 Department of Neurosurgery, Haaglanden Medical Center, The Hague, The Netherlands; 5 Department of Neurosurgery, Leiden University Medical Center, PO Box 9600, 2300 RC Leiden, The Netherlands; 6 Department of Neurology, Massachusetts General Hospital, Boston, MA USA

**Keywords:** Leptomeningeal metastases, Hydrocephalus, Ethics, Shunt, Quality-of-life

## Abstract

**Purpose:**

Leptomeningeal metastases (LM) are a rare, but often debilitating complication of advanced cancer that can severely impact a patient’s quality-of-life. LM can result in hydrocephalus (HC) and lead to a range of neurologic sequelae, including weakness, headaches, and altered mental status. Given that patients with LM generally have quite poor prognoses, the decision of how to manage this HC remains unclear and is not only a medical, but also an ethical one.

**Methods:**

We first provide a brief overview of management options for hydrocephalus secondary to LM. We then apply general ethical principles to decision making in LM-associated hydrocephalus that can help guide physicians and patients.

**Results:**

Management options for LM-associated hydrocephalus include shunt placement, repeated lumbar punctures, intraventricular reservoir placement, endoscopic third ventriculostomy, or pain management alone without intervention. While these options may offer symptomatic relief in the short-term, each is also associated with risks to the patient. Moreover, data on survival and quality-of-life following intervention is sparse. We propose that the pros and cons of each option should be evaluated not only from a clinical standpoint, but also within a larger framework that incorporates ethical principles and individual patient values.

**Conclusions:**

The decision of how to manage LM-associated hydrocephalus is complex and requires close collaboration amongst the physician, patient, and/or patient’s family/friends/community leaders. Ultimately, the decision should be rooted in the patients’ values and should aim to optimize a patient’s quality-of-life.

## Introduction

Leptomeningeal metastases (LM) are being increasingly encountered in present-day clinical practice. This greater incidence is likely due to advances in the treatment of cancer that allow patients to live long enough to develop late complications such as LM [1–3]. Yet, despite the progress in oncologic treatments, the prognosis for LM remains poor with average survival ranging from 3.5 to 6 months [1–4].

LM affects approximately 5–8% of patients with solid tumors [5]. Patients with LM typically present with acute and often debilitating neurologic symptoms, including headaches, nausea, cognitive dysfunction, seizures, cranial nerve deficits, and/or weakness and pain [2, 4]. As these symptoms greatly affect patients’ quality-of-life [1], it is of importance to effectively manage them. Hydrocephalus (HC) secondary to LM is common [6], though outcomes in patients with metastases-related hydrocephalus are poor with approximately 10% 1-year survival [4].

Management of hydrocephalus in patients with LM is challenging, as effective, immediate symptom relief often requires surgical intervention in frail cancer patients [4]. Although the available data exploring the effectiveness of ventriculoperitoneal shunt (VPS) placement in improving quality-of-life in this patient population is limited, the procedure is commonly performed to relieve patients of hydrocephalus-associated symptoms [1].

There is currently no clear medical consensus for the best treatment option to manage LM-associated hydrocephalus. We believe this question is less of a medical question and more of an ethical one. Given that the prognosis for patients with LM can vary based on performance status, systemic disease control, age, and primary tumor histology [3, 7], we believe that a more nuanced, patient-centered approach to managing their hydrocephalus should be undertaken. This approach should look not only at the hard facts surrounding expected disease course, but combine patient quality-of-life measures, cultural values, and fundamental ethical principles in determining the ideal management option for these patients.

Here, we aim to briefly present the current surgical options for LM-associated hydrocephalus and the pros and cons surrounding each. Subsequently, we will offer ways to facilitate physician and patient decision-making based on general ethical principles applied to patients with LM-associated hydrocephalus.

## Current surgical options

Current options for management of LM-associated hydrocephalus include: (1) shunt placement; (2) repeated lumbar punctures; (3) intraventricular reservoir placement; (4) endoscopic third ventriculostomy; or (5) no intervention with focus on pain management [3, 4].

### CSF diversionary procedures: ventroperitoneal or lumbar shunting

VPS involves placement of a catheter in the lateral ventricle, connected into the peritoneal cavity [8]. VPS allows for rapid normalization of elevated intracranial pressure (ICP) and is thus commonly employed to relieve symptoms of hydrocephalus [5, 8, 9]. Table 1 depicts studies that have previously reported symptoms and outcomes for patients with leptomeningeal disease-associated HC who have undergone shunting. Overall, these retrospective series have demonstrated significant improvement in hydrocephalus-associated symptoms following VPS placement, with rates of symptom improvement ranging from 75 to > 90% (Table 1) [4, 5, 9–11]. Shunting has also led to marked patient improvements in performance status, with post-surgical Karnofsky Performance Status (KPS) increases of 10–20 points [4, 11]. While it has not been standard-of-care to intervene on patients with low performance scores, the minimal published data suggests that VPS may improve quality-of-life even in patients with low KPS [9, 10].

**Table 1:** Characteristics and outcomes of patients treated for leptomeningeal disease-associated hydrocephalus

Study	Primary pathology (n)	No. of patients	Presenting symptoms (n)	Intervention (n)	Symptom improvement, overall rate	Symptom improvement by symptom (n)	Complications overall %; type (n)	Perioperative mortality	Median survival from LMD diagnosis (months [OQR])	Median survival from surgery, [IQR]
Lokich et al. [14]	Lung adenocarcinoma (2), breast (1)	3	Gait issues (3), headache (2), nausea (1), cognitive changes (1)	VPS (3)	3/3 (100%)	Gait issues (3), headache (2), nausea (1), cognitive changes (1)	N.R.	0/3 (0%)	N.R.	6 months (4-7.5 months]
Omuro et al. [5]	Breast (23), lung (6), melanoma (3), other (5)	37	Headache (24), nausea/vomiting (18), cognitive changes (19), gait issues (20), spinal symptoms (10), hemiparesis (12), papilledema (4)	VPS (37/37)	27/37 (77%)	Most had decreased headache, nausea/vomiting, and improved level of alertness^a^	11%; shunt malfunction requiring revision (3), subdural hematoma (1), infection (0), peritoneal carcinomatosis (0)	0/37 (0%)	4 [3 days to > 3.6 years]^b^	2 months [2 days to > 3.6 years]^b^
Lee et al. [11]	NSLC (29), SCLC (2), mixed NSLC/SCLC (1), breast (8), colorectal (3), renal cell (2), other (5)	50^c^	Headache (35), cognitive changes (17), gait issues (7), urinary incontinence (5), seizures (2)	VPS (50/50)	40/50 (80%)	Headache (30), cognitive changes (12), gait issues (5), urinary incontinence (2), seizures (2)	8%; Valve malfunction (1), increased ICP (1), overdrain (1), uncontrolled hydrocephalus (1), infection (0), peritoneal carcinomatosis (0)	1/50 (2%)	3.5 [0–28]^b^	3 months [2 days–54 months]^b^
Gonda et al. [10]	Lung (13), melanoma (10), breast (9), renal (3), colon (1)	36^d^	Headache (6); headache and nausea (10); headache, nausea, vomiting (8); headache, lethargy (7); headache, neurological deficit (5)	VPS (36/36)	27/36 (75%)	Headache (3); headache and nausea (8); headache, nausea, vomiting (8); headache, lethargy (7); headache, neurological deficit (1)	19%; wound infection (4), shunt occlusion (2), hygroma (1), peritoneal carcinomatosis (0)	0/36 (0%)	N.R.	90 days [47–99 days] for LMD patients only
Jung et al. [24]	NSCLC (37), SCLC (8), breast (14), gastric (4), other (8)	7^e^	N.R.	VPS (7/7)	N.R.	N.R.	N.R.	N.R.	N.R.	5.7 months [95% CI 0.000–13.173 months]
Yamashiro et al. [1]	Lung adenocarcinoma (4)	4	N.R.	LPS (4)	3/4 (75%)	N.R.	N.R.	0/4 (0%)	N.R.	7.5 months [4.5–15 months]
Murakami et al. [4]	Lung (4), breast (4), ovarian (1), colon (1), nasal rabdoidsarcoma (1)	11	Headache (10), nausea/vomiting (6), cognitive changes (6), gait issues (4)	LPS (3), VPS (8)	10/11 (91%)	Headache (9), nausea/vomiting (5), cognitive changes (5), gait issues (1)	9%; shunt obstruction requiring revision (1), infection (0), peritoneal seeding (0)	1/11 (9%)	3.9 [3.5–6.3]	3.3 months [2.9–5.7 months]

*N.R*. not reported, *LPS* lumboperitoneal shunt, *VPS* ventroperiotneal shunt

^a^Specific numbers for each symptom not reported

^b^Range (minimum to maximum survival)

^c^Includes 10/50 patients with parenchymal, non-LMD associated hydrocephalus

^d^Includes 27/36 patients with parenchymal, non-LMD associated hydrocephalus; only 9 had LMD-associated hydrocephalus but outcomes not reported separately

^e^Study included 71 patients, but only 18 had LMD-associated hydrocephalus, of whom only 7 underwent shunting

Despite the symptomatic relief following VPS experienced by many patients, there are gradations of benefit based upon various patient factors. For example, it appears that while headache and nausea are most likely to improve following VPS, results are variable with respect to cognitive dysfunction, urinary incontinence, and gait dysfunction [9, 11]. Moreover, per the results of Murakami et al., only patients with a presenting KPS ≥ 30 benefited from VPS. In contrast, those with a KPS < 30 before surgery died within 1 month of their procedure due to worsening of their general condition [4]. In addition, while prior studies have shown that in general, patients with LMD from breast cancer, leukemia, and lymphoma tend to have slightly improved survival compared to LMD from other primary tumor sites [12, 13], the studies in Table 1 that focused on patients with LMD-associated hydrocephalus did not find primary tumor histology to be prognostic for survival [5, 11]. However, the improvement in symptoms following VPS did allow for subsequent administration of systemic therapies in some cases, possibly contributing to increased survival [1, 11]. These results suggest that in patients with primary tumors for which systemic therapies are available, alleviation of debilitating symptoms via placement of a shunt may allow for more rigorous treatment.

VPS also poses potential risks, including infection, bleeding, and peritoneal tumor dissemination, although none of the studies in Table 1 found any cases of peritoneal carcinomatosis after VPS [1, 5]. These studies have demonstrated complication rates between 9 and 19% [5, 9, 10], often leading to a second surgery for shunt revision [4, 5, 11].

As seen in Table 1, the data on outcomes following VPS in patients with LMD is sparse, and there is thus no obvious paradigm for clinicians to follow when recommending shunt surgery for LM-associated hydrocephalus. Even after VPS, median survival across studies remains low, ranging from 2 to 7.5 months (Table 1). Only one prior study had a “non-surgical” group to compare to the shunted patients, and while there was improved survival in the group that underwent shunting, this difference was non-significant, and there was also no data on symptom relief or quality-of-life to compare across the two groups. Thus, while those with severe presenting symptoms of headaches and nausea may derive the greatest benefit, more conservative approaches may be suitable for those with cognitive symptoms that are unlikely to improve or those with very low KPS. In addition, in the studies that did report on complications, the risks were non-negligible, and often led to revision surgery, which might pose significant challenges in a population of patients that is seriously ill and with poor reserve to begin with.

### Repeated lumbar punctures or placement of intraventricular reservoir

Other potential options for relieving elevated ICP secondary to hydrocephalus involve repeated lumbar punctures (LPs) or the placement of an intraventricular reservoir [3, 15]. While typically performed to obtain CSF for diagnostic purposes, LPs have also been shown to result in rapid improvement of acute symptoms secondary to hydrocephalus [8, 16]. However, serial LPs are not ideal as they subject patients to the constant discomfort of repeated intervention.

Intraventricular reservoirs, in contrast, work similarly to LPs, but are less imposing on patients. Such reservoirs are placed underneath the scalp in the frontal horn of the right lateral ventricle; a connecting catheter allows for communication between the device and CSF. Generally used for delivery of intrathecal chemotherapy in patients with leptomeningeal disease (LMD), they can also allow for CSF aspiration in the setting of high ICP [15, 17]. Compared to LPs, repeated access to CSF is easier and more comfortable for patients with a reservoir.

However, reservoirs also come with possible complications, including infection, hemorrhage, or malpositioning [8, 17]. Malpositioning rates range from 2.7 to 12.5% and can have serious consequences [8, 17, 18]. If a patient with hydrocephalus is not receiving chemotherapy, the utility for such an intervention compared to more permanent solutions must be weighed against the burden of an additional procedure that may eventually require conversion to a more sustainable solution anyways.

### Endoscopic third ventriculostomy

LMD can present with either obstructive or non-obstructive hydrocephalus [5]; while VPS remains the preferred treatment in patients with non-obstructive hydrocephalus, endoscopic third ventriculostomy (ETV) may be another potential therapeutic option in those with obstructive hydrocephalus [17, 19]. ETV involves the endoscopic placement of a small hole at the floor of the third ventricle that subsequently allows for diversion of CSF into the interpeduncular cistern [17, 20]. It is minimally invasive, has a low operative morbidity, and is generally less uncomfortable for patients compared to VPS [19, 21]. LMD impairs CSF resorption capacity, and ETV would therefore not be expected to be efficacious in such cases of communicating HC [19]. The use of ETV in patients with LMD-associated hydrocephalus has therefore not been well-studied, although a few reports have looked at its role in patients with hydrocephalus secondary to parenchymal brain metastases [10, 19, 21]. These studies have demonstrated symptom improvement rates of ~ 70% in these patients, with lower or similar complication rates as compared to those following VPS [10, 19, 21].

One study triaged patients with hydrocephalus secondary to cerebral metastasis to either VPS or ETV based upon whether they had non-obstructive or obstructive hydrocephalus, respectively, and showed similar clinical outcomes in terms of symptom palliation, post-operative KPS improvement, and overall survival between the two groups [10]. While the difference in the overall complication rate between the two groups did not reach statistical significance, VPS patients had a greater complication rate of 19.4 versus 12.6% in the ETV group [10]. These results suggest that in patients with metastasis-related, obstructive hydrocephalus, the less invasive option of ETV might offer a similar degree of benefit to patients as VPS, but without as many complications. While the aforementioned studies did not assess a role for ETV in patients with LMD, their results suggest ETV may be an effective option when hydrocephalus is secondary to bulky LMD impairing CSF outflow [5]. Each patient’s radiographic abnormalities should thus be studied to determine the cause of the hydrocephalus and subsequently to determine whether ETV might be a more suitable option than VPS in these patients. Overall, further study of ETV in patients with LMD is warranted.

### Non-intervention

Patients may also opt not to treat their hydrocephalus. While the above studies demonstrate the effectiveness of surgical intervention on immediate symptom relief, the prognosis even after treatment of metastasis-related hydrocephalus remains poor [9]. Thus, the burden of an additional intervention may not be desirable. Median survival in patients with untreated LM ranges from 1 to 2 months, sometimes extending to 6 months in aggressively-treated populations [21–24]. While many of these patients die from progression of their systemic cancer [25], per some reports in the literature, the majority of deaths in this patient population occur due to progressive neurological involvement of the patients’ disease [22, 23], underscoring the importance of determining whether neurosurgical intervention is justified if it is the neurological sequelae that are leading to patient death. A study by Jung et al. explored the prognostic significance of surgically treated hydrocephalus in LM and found improved overall survival in surgically treated hydrocephalus as compared to surgically untreated hydrocephalus (median 5.7 versus 1.7 months), although this did not reach statistical significance [24]. While this study did not evaluate quality-of-life or quality-of-death, studies should assess the comfort and pain levels of patients who live longer following surgical intervention for hydrocephalus. This information could allow clinicians and patients to better weigh the risks and benefits of intervention versus non-intervention.

### Ethics of intervention

The lack of clear medical consensus on how to manage LM-associated hydrocephalus likely stems from a lack of robust data, as well as wide variation in patient presentation, belief systems, and environmental resources. Together, these make it difficult to recommend a general paradigm applicable to all patients. Patients’ prognoses differ based upon prior and concurrent treatments, performance status, systemic disease burden, primary tumor histology, and other factors [3, 7]. Still, most patients with LM tend to be seriously ill and generally have poor prognoses. This complicates the decision to actively intervene at the risk of exposing the patient to further complications versus focusing on comfort measures alone.

As mentioned above, there is data to suggest prolonged survival following treatment of LM-associated hydrocephalus [24]. However, assessment of patient QOL during this extended survival period is lacking. Moreover, given that even after the surgery, median survival is still on the order of months, we think that fundamental ethical principles as opposed to statistics can better guide decision-making.

Beauchamp and Childress have previously proposed a four-principles approach to medical ethics, highlighting the following ethical principles: respect for autonomy; non-maleficence; beneficence; and justice [26]. Below, we will briefly define these principles and apply them to LM-associated hydrocephalus.

### Respect for a patient’s autonomy

Respect for autonomy means that the physician accepts the choice made by a patient with decision-making capacity [26]. As patients with LM may have poor performance status and possibly neurocognitive sequelae [2], it is essential that the decision-making capacity of the patient is assessed early. Moreover, patients with LMD are generally terminally ill and comprise a particularly vulnerable group of individuals [27]. Some have previously suggested that decision-making capacity is impaired in patients who are nearing death, making true informed consent particularly challenging to obtain [28, 29]. Furthermore, terminally ill patients often feel a sense of desperation stemming from unbearable symptoms and a hope to try any last effort to relieve suffering. Such desperation limits the voluntariness of any major decision-making [28, 30].

Thus, before the various options for management are even discussed with the patient, the physician should assess his or her competence. If the patient demonstrates full decision-making capacity, it is up to the team to clearly explain each of the above possible interventions, as well as their expected short-term and long-term consequences, so that the patient can make an informed decision. If a patient does not demonstrate decision-making capacity, a proxy can be used to make decisions on their behalf [29, 31]. When no proxy has been named, these decisions are typically left to family members, physicians, and hospitals, who are expected to practice “substituted judgment,” i.e. to make a decision as the ill individual would have if they had been able to decide themselves [32].

Since each of the above options for LM-associated hydrocephalus will have different consequences for the patient’s life, the patient’s personal values should dictate the course-of-action. Therefore, it is essential to assess the capacity of the patient and ensure that either the patient, if capable, or someone familiar with the patient’s belief systems be at the forefront of the decision-making process.

Studies have shown that patients’ values and religion may have particularly strong influences on end-of-life planning. For example, Hindus and Buddhists typically prefer dying at home, which may ultimately dictate what types of treatments they choose to receive [33]. To illustrate, if the placement of a shunt allows a patient to return home because they no longer have uncontrollable nausea requiring a nasogastric tube, that patient may choose to undergo shunting even if it means they will have to undergo a potentially risky surgical intervention.

Physicians treating patients with LM face challenging decisions. They should first prioritize establishing patient capacity, and once established, they should show utmost respect for the patient’s autonomy, rooted in his or her personal values, religious systems, and/or cultural paradigms. Through a shared decision-making approach that brings together medical information from the physician with the belief systems inherent to the patient, an optimal plan individual to that patient can be reached.

### Non-maleficence

Non-maleficence means that physicians have the obligation to avoid harm to their patients [26, 34]. Omitting a standard-of-care and exposing a patient to undue harm violates the principle of non-maleficence [34]. We believe that it is the responsibility of the physician and patient to work together to determine the course of action that minimizes harm to the patient while staying true to the patient’s own values, as described above.

Depending on the patients’ beliefs, he or she may favor relief in the short-term over long-term suffering, while others may view this as burdensome prolonging of life (or even suffering). Such views on what course of action is “less bad” are often influenced by patient religion and culture. For example, in Roman Catholicism, “Natural Law,” is used to determine how to act morally in complex situations that may have both good and bad consequences [33]. Overall, an action with a negative consequence may be allowed if some greater good also occurs as a result of the action. For example, shortening of life may be permissible if it allows for alleviation of suffering. In the context of shunting in LM, such a belief system may prompt a patient to choose not to undergo intervention and opt for increasing pain medications instead, even if this means hastening death [33].

The theme once again emerges that in our case of leptomeningeal metastases, the decision of whether or not to intervene is unclear. However, what is clear is that a discussion with the patient and what he or she views as more or less “bad” should be undertaken to uphold the principle of non-maleficence and minimize harm to the patient, whether that be in the form of present-day or later suffering.

### Beneficence

Per the principle of beneficence, physicians have an obligation to benefit their patients and to balance these benefits against any risks [26, 34]. Given the relative sparsity of data on quality-of-life measures following treatment for LM-associated hydrocephalus, upholding the principle of beneficence in such patients involves open discussion of all options, consideration of each patient’s individual prognosis, as well as the patient’s overall hopes and goals for any treatment. A patient with a KPS of 20 who is too weak to withstand another intervention, is unlikely to benefit from surgery. On the contrary, a young patient with a KPS of 70 who wishes to play an active role in their children’s lives but is debilitated by headaches and nausea would likely benefit from intervention. Beneficence, or what is best for one patient, may be different from what is best for another; thus, physicians should follow an individualized course for each patient that optimizes his or her health and comfort while minimizing any associated harms.

### Justice

The fourth principle of biomedical ethics is justice, which pertains to ensuring fairness in how health benefits are distributed in society [26]. While this principle has typically been explored in the context of situations in which there is a scarcity of resources (e.g. organ transplantation, rationing of intensive care unit beds [35]), we think it is applicable in any medical setting.

Numerous studies have demonstrated that physicians may exhibit implicit bias against certain patient populations based on race, sex, or other characteristics, and that this implicit bias has been linked to poorer communication, lower quality care, and differential treatment of these groups [35–38]. While not directly related to the allocation of tangible medical resources, such disparities and inequities in patient care violate the principle of justice.

Extrapolating this data to our case, it is important for physicians to maintain the same thoroughness in communication with all LM patients, regardless of race, sex, or education-level. In fact, attention to justice may be even more critical in cases involving LM, where the pathophysiology is particularly complicated and may be difficult for physicians to explain to laypersons. By leaving out information for some patients and thereby preventing those patients from having all the necessary data to make a decision, the physician would be violating the principle of justice. Thus, we believe that a key piece of the decision-making paradigm in how to manage LM-associated hydrocephalus requires the physician to spend ample time with and to equip each patient with a thorough enough understanding from which to make a decision.

### Shared decision making

Ultimately, a decision as complex as how to manage LM-associated hydrocephalus requires open discussion and collaboration amongst the patient, patient’s family/close friends, and physician. First and foremost, we believe that the physician needs to establish the decision-making capacity of the patient. Once clear who the decision-maker for the patient will be (either the patient or a proxy), the physician should communicate all options to this individual and any other individuals whom the patient wishes. It is the responsibility of the physician to be complete and unbiased in presenting the objective risks and benefits of each option, tailored to that patient’s individual prognosis and disease presentation. Once this “factual” baseline is established, the physician should learn the patient’s values, cultural beliefs, and personal priorities. In situations where it may be helpful for the patient, religious or community leaders may also be involved to weigh in on the decision.

Recently, there has also been an increasing amount of evidence that supports the early involvement of palliative care teams in the care of cancer patients [39]. In those with terminal illnesses, these teams are specially trained to focus on caring for patients in a way that aligns with their values. Moreover, palliative care specialists, because of their interdisciplinary nature, are optimally positioned to facilitate communication among the many teams taking care of cancer patients [39]. Indeed, data from multiple randomized controlled trials have demonstrated that when palliative care is integrated with routine oncological care, patients report improved quality-of-life, less aggressive end-of-life care, reduced symptom severity, and improved prognostic awareness [38–42]. Based on these promising reports, palliative care physicians should be increasingly integrated into this shared decision-making model, as well.

With both the objective data set forth by the physician and the belief system of the patient made apparent, the two should work with teams like the palliative care doctors to re-assess the options and finally come to a shared decision. This shared decision should aim to respect the patient’s autonomous choices, while also staying true to the ethical medical principles of doing good and minimizing harm. By listening to and engaging with their patients, physicians can prioritize the patient’s dignity and comfort at a time of deep suffering and pain.

## Conclusions

LM-associated hydrocephalus severely impacts length and quality of life. Given the paucity of quality-of-life and survival data, there is currently no consensus in the medical community regarding how best to manage hydrocephalus in these patients. Treatment options include (1) shunt placement; (2) repeat lumbar punctures; (3) intraventricular reservoir placement; (4) endoscopic third ventriculostomy; or (5) no acute intervention with a focus on pain management. We believe that the four ethical principles put forth by Beauchamp and Childress (autonomy, non-maleficence, beneficence, and justice) can aid in analysis of how to manage LM-associated hydrocephalus. Furthermore, we believe that the management of LM-hydrocephalus should hinge upon rigorous upholding of these principles as well a highly individualized, patient-centered discussion of the management options. In this way, patients with a serious illness can safely and strongly live out whatever time they have left in a matter consistent with their personal and familial values.
